# Animal health and welfare as a public good: what do the public think?

**DOI:** 10.1007/s10460-024-10585-0

**Published:** 2024-05-30

**Authors:** B. Clark, A. Proctor, A. Boaitey, N. Mahon, N. Hanley, L. Holloway

**Affiliations:** 1 Centre for Rural Economy, School of Natural and Environmental Sciences, Newcastle University, Newcastle upon Tyne, NE1 7RU UK; 2 School of Environmental Sciences, University of Hull, Cottingham Road, Hull, HU6 7RX UK; 3 The James Hutton Institute, Craigiebuckler, Aberdeen, AB15 8QH Scotland, UK; 4 School of Biodiversity, One Health and Veterinary Medicine, University of Glasgow, Glasgow, G12 8QQ Scotland

**Keywords:** Animal health, Animal welfare, Public goods, Public perceptions

## Abstract

This paper presents a novel perspective on an evolving policy area. The UK’s withdrawal from the EU has led to the creation of a new Agriculture Act and proposals for significant changes to the way farming subsidies are structured in England. Underpinned by a ‘public money for public goods’ approach, where public goods are those outputs from the farm system which are not rewarded by markets, yet which provide benefits to many members of society. New schemes include the Animal Health and Welfare Pathway, where certain aspects of farm animal health and welfare (FAHW) will be subsidised through government support, raising a much-debated issue in the literature regarding the representation of FAHW as a public good. For policy to be responsive to societal demands and accountable to citizens, understanding public attitudes and preferences towards FAHW as a public good, and how the public might prioritise this in relation to a wider suite of environmental public goods from farming, is important. An online survey of 521 members of the UK public was conducted and analysed with descriptive statistics and ordered logistic regression. Findings reveal low awareness of the changing agricultural policy context, but strong support for public money being used to provide public goods, particularly for FAHW. Findings also indicate a need for more effective public communication of farming and FAHW issues from farming stakeholders to ensure public policy in this domain is responsive and accountable to its citizens. Further work is needed to inform future debates and engagement surrounding FAHW, including through which combination of funding mechanisms (public or private) it is provided.

## Introduction

The UK’s withdrawal from the European Union (EU) has necessitated the development of its own independent agricultural policy (Siettou 2022). Whilst the devolved nations of Scotland, Northern Ireland and Wales are developing their own approaches, in England this has led to considerations surrounding if and how farm subsidy payments should be made. This has led to a shift in approach from area-based and environmental payments currently supported through Pillars 1 and 2 of the EU Common Agricultural Policy (CAP), towards a system mainly structured around a ‘public money for public goods’ approach (Hejnowicz and Hartley 2018), where public goods are understood as those which are non-rival and non-excludable in consumption which the market fails to reward farmers for producing, yet which are valued by multiple members of society (Krugman and Welles 2015). Whilst the EU is not planning on withdrawing area-based payments, payments for public goods are highlighted within the 2020 EU Farm to Fork Strategy and 2023–2027 CAP (European Commission, no date, European Commission 2020) which emphasises strategic objectives linked to social, environmental and economic sustainability tied to associated payment schemes (European Commission, no date). There is also evidence of discussions surrounding the funding of public good delivery from farming in other parts of the world, such as the United States (Shortle and Uetake 2015).

In England, the Department for Environment, Food and Rural Affairs (Defra) has set out this new approach to farmer support through the *Future Farming Programme*, which includes a series of environmental land management (ELM) schemes (Defra 2021). Under ELM, land managers will be paid to deliver a range of environmental public goods including greenhouse gas mitigation, water and soil conservation, and wildlife and biodiversity enhancement. Running in parallel are proposals for how public funds could be used to deliver farm animal health and welfare (FAHW) enhancements that are currently not sufficiently delivered by the free-market (Defra 2022a), through an Animal Health and Welfare Pathway (AHWP). Private funding for such public good delivery is also being encouraged (House of Commons 2022). This evolving policy context in England reignites a wider debate around the notion of FAHW as a public good.

Improving FAHW delivers both private (higher income for farmers through healthier animals, higher mental well-being for farmers) and public benefits (for example, consumers may care about farm animal welfare, whilst reductions in disease delivers benefits for other stakeholders). Improving FAHW has been on the EU and UK political agenda for decades (Fraser 2008). Whilst improved FAHW has been associated with a range of benefits including increased food quality and lower greenhouse gas emissions from the dairy sector (Rushton et al. 2007; Cooper et al. 2009), delivering enhanced FAHW has long been recognised as imposing costs on primary producers (The Brambell Report 1965) through changes to production methods and increased recording costs (Sørensen et al. 2007), which may act as a disincentive for farmers to increase their spending on FAHW. These costs either need to be accounted for through targeted subsidies, or recouped via the market through increased product prices, where labelling is used to signal these qualities to consumers (Lusk and Norwood 2011). However, the latter is dependent on consumers being willing and able to pay higher prices and having sufficient information on FAHW to make informed decisions as to the products they are choosing between (Verbeke 2005; Kehlbacher et al. 2012; Heise and Theuvsen 2017).

Given the significant taxpayer costs associated with farm subsidies, understanding public support for their provision is imperative if they are to be socially accepted. Whilst the development of proposed subsidy changes in England has emphasised co-design with farmers and other experts (Defra 2020b), the wider public has so far had limited input into debates about the delivery and financing of specific public goods (Defra 2018). Assessing whether people support these reforms is important (Howley et al. 2014), especially in their role as consumers (Vanhonacker and Verbeke 2014). This is particularly pertinent since a key premise of the concept of food democracy is for public policy to be responsive and accountable to its citizens (Lang 1999), with failure to do so thought to threaten the legitimacy of associated regulatory frameworks and agencies (Frewer et al. 2004).

Research has consistently shown evidence of public concern over FAHW (Clark et al. 2016; Eurobarometer, 2015), with several studies exploring if and how this concern varies across different population groups and might translate into a perceived consumer responsibility through an enhanced willingness-to-pay (WTP) in the marketplace (Lagerkvist and Hess 2011; Clark et al. 2017; Rodrigues and Hanley 2021). However, little is known about how this may translate into public support for farm payment-based approaches specifically aimed at FAHW (Defra 2018). Understanding public perceptions surrounding farm payments is therefore important in considering where responsibility for FAHW is thought to lie and who should pay for it, and there is a need to establish whether consumers indeed perceive FAHW as a public good. This paper addresses these questions by examining how the public prioritises FAHW in relation to other outcomes of agricultural systems that could be public goods, and where they think responsibility for FAHW should lie. Building on work exploring public WTP for FAHW (Rodrigues and Hanley 2021), this research uses a survey-based approach and aims to highlight public perspectives and understandings in contributing to this evolving policy space and considers the implications of the findings for wider discussions around FAHW as a public good.

## Public goods, public perceptions and farm animal health and welfare

### Public goods from agriculture

Although originally developed and used in economics, the term “public good” has more recently been adopted and adapted by policy makers as an approach underpinning the redesign of farm subsidies for the delivery of public benefits (Gravey 2022). From an economics perspective, public goods are those that are considered to be both non-rival (if one person uses it, it does not stop others using it, or reduce the quantity/quality of that good available to others) and non-excludable (once the good is supplied it is virtually impossible to exclude others from consuming it, whether they pay for it or not) (Krugman and Welles 2015). Whilst very few goods are entirely non-rival or non-excludable, policy-relevant public goods exhibit varying degrees to which they fulfil these criteria (Kipling 2019).

The idea of public goods is closely linked to that of “externalities”. The production or consumption of some goods generates additional costs or benefits to third parties not involved in the original transaction, and these side-effects are known as externalities (Lusk 2011). Externalities can be either positive (generating additional benefits) or negative (generating additional costs – financial or otherwise). Externalities are always outside of the usual market mechanisms of payment and so are not accounted for in market transactions (Hubbard et al. 2020). When externalities exist, the market price of a product will not reflect the full social costs (or benefits) of production, and consumers will consume too much (or too little) of the good from the viewpoint of social welfare (Lusk 2011; Hubbard et al. 2020). When farmers fail to invest in adequate biosecurity, for example, they can impose disease transmission costs on other producers – an example of a negative externality, or external cost.

This inability of the market to produce what society values the most contributes towards market-failure (Krugman and Welles 2015). When market-failure occurs, and the goods are deemed valuable to society (i.e., they are desirable public goods), government intervention is typically needed (Rushton et al. 2007). Such intervention can take the form of government taking responsibility for providing them (e.g. providing publicly-funded vets), or by financially encouraging others to provide them, e.g., via subsidies generated through tax revenues, or taxes imposed on external costs such as pollution, or through creating regulated markets in rights to such goods.

Whilst a core role of agriculture in capitalist societies is to produce private goods for sale (i.e., human food, animal feed, fibre and energy), farming has a long history of contributing public goods (Meijboom and Stafleu 2016). Certain environmental goods have previously been categorised as having public benefits (Nègre 2021), with the EU CAP reforms of 1992 and 2000 in particular supporting this agricultural ‘multifunctionality’ (Dwyer et al. 2015). The term multifunctionality, coined in the late 1980s, describes how land managers can produce a variety of outputs including natural and socio-cultural benefits alongside private goods. It has subsequently been superseded in policy circles by the term public goods, which has further facilitated the shift of farm support payments towards the delivery of wider environmental and social benefits (Dwyer et al. 2015).

Agricultural public goods can generally be classified into two main groups and their associated externalities: environmental goods (e.g., biodiversity, water quality, climate change mitigation) and social goods (e.g., food safety and animal welfare) (Howley et al. 2012). Whilst there would appear to be strong public demand for the provision of social goods provided by the agricultural sector (*inter alia* Arriaza et al. (2004), it may be difficult for producers to recoup the costs incurred through their provision via market-based mechanisms such as price premiums for higher welfare products (HWP). Subsequently, without appropriate intervention, farmers may be unable or unwilling to supply a sufficient quantity of these public goods (Cusworth and Dodsworth 2021). This market-failure has meant that several governments have sought to support farmers to deliver public goods (Buller and Morris 2004). Across Europe, the key mechanism for this historically has been through the CAP, and specifically Pillar Two agri-environment-climate schemes. More recently, in post-Brexit England, this has been integrated into new schemes including ELM and the AHWP.

### Contesting FAHW as a public good

The notion of FAHW^1^ as a public good is contested^2^, and an extensive commentary exists surrounding whether it should be treated as such (Hubbard et al. 2020; Harvey and Hubbard 2013; Nurse 2016; British Veterinary Association 2018), with arguments both for and against its inclusion in this category (Bateman and Balmford 2018; Mann 2005; Gard 2018).

Animal (health) management involves a complex consideration of ethical principles and animal wellbeing (Perry et al. 2001), as well as economic factors (including productivity) and human and environmental health. Poor animal welfare is considered a ‘public bad’ for humans and the environment (Cox and Bridgers 2019), not to mention negatively effecting the animals themselves (Lusk 2011). Conversely, higher standards of animal welfare, as well as improving animals’ lives, can provide the additional benefits to society typical of public goods (Nurse 2016). For example, Cooper et al. (2009) highlight that good husbandry practices contribute to enhanced consumer safety and public health. These highlight the wider social and environmental benefits from improvements to FAHW, including for non-consumers (e.g., vegans and vegetarians) who may still have an interest in how animals are raised (Bennett et al. 2012), yet do not directly express their preferences for particular animal production practices through their purchases. In addition, there are ethical considerations surrounding the use of animals for food given animals’ own intrinsic value (Christensen et al. 2012), as well as their economic value as a resource in food production (see McInerney (2004). These externalities may also be generated through the subjective perceptions of animal suffering (Uehleke and Hüttel 2019). Indeed, it is stated that,

“…the non-market or ethical aspects of animal welfare therefore demand attention by government of how best to deal with animal welfare as a public good…” (Lawrence and Vigors 2020) p18).

FAHW can be promoted both through legislation and market-based approaches (Sørensen and Schrader 2019). Whilst government legislation has traditionally been the main method for improving standards within farm animal production systems within the EU and UK (Bennett 1997), those who argue *against* FAHW as a public good highlight the role of the private sector in delivering acceptable, or higher than minimum, animal welfare standards (Sandøe and Christensen 2018; Grethe 2017). This has been the case in the United States, where welfare developments have often been in response to private initiatives reacting to increased public awareness or changing consumer demand (Dana and Nadler 2019). Some argue that the private sector needs to demonstrate that it can effectively and efficiently provide desired levels of FAHW (Rushton et al. 2007), in order to maintain a social licence to operate (Leith et al. 2014). However, the higher/premium price for HWP fails to capture all of consumers’ WTP, with a lack of knowledge or relevant information at the point-of-purchase (defined as information asymmetry in economics), competing interests and decision-making context contributing factors (Hubbard et al. 2020; Norwood and Lusk 2011).

Whilst in economics public goods are defined by their non-rival and non-excludable properties (Krugman and Welles 2015), in political science the term is not linked to these characteristics, instead being focused on ‘what is good for people and what people want for their collective well-being’ (Dwyer et al. 2015), p5). As such, public goods are what is collectively or institutionally in the public interest or utility (Dwyer et al. 2015). The 2020 UK Agriculture Act acknowledges that farms should be treated like any other sector of the economy given they produce products for private consumption, whilst also recognising that they produce more than just food, i.e., they have a multifunctionality, so any additional funds should be directed at supporting this element (Gravey 2022). The inclusion of some aspects of FAHW in future farming proposals in England indicates an interesting potential policy shift towards acknowledgement of FAHW as a public good.

### The public’s role in influencing FAHW as a public good

If FAHW is to be defined as a public good then some have suggested that the public should also have a role in deciding if and how these goods should be supported (Kaul and Mendoza 2003). This follows the political science perspective on public goods (Dwyer et al. 2015) which suggests the public should have a say on what is of interest or value to them. Further, it links with the premise of food democracy, with a requirement for policy to be responsive to societal perceptions and accountable to all citizens (Hassanein 2003). It also aligns closely with the principles of welfare economics, and specifically with cost-benefit analysis, wherein consumer preferences are the key indicator of societal benefits from changing policy (Hanley et al. 2009).

Thus, understanding public preferences is important (Howley et al. 2014), and particularly pertinent given the notable levels of public concern over FAHW (European Commission 2016). A slowdown in European FAHW regulation since the early 2000s has resulted in market-based solutions being increasingly used to facilitate improvements in welfare standards in response to public concerns^3^. This is typically through quality assurance labels or brands (Sandøe and Christensen 2018) given that welfare standards cannot be identified from the product itself due to their credence nature (Grunert et al. 2004), i.e., they are a quality that cannot be observed by the consumer even after purchase, instead relying on information provided by others to aid these decisions (Grunert et al. 2000). Whilst research has shown that consumers might say they have a WTP for improved FAHW (Clark et al. 2017; Lagerkvist and Hess 2011), this varies by several consumer characteristics and does not always correspond however to actual purchasing behaviour (Clark et al. 2016). Reasons behind this include a lack of information and disconnect from farming by consumers, an inability to identify HWP, and other competing interests including price and time constraints (Clark et al. 2016). This suggests an inability of competitive markets to both achieve and deliver societally optimal levels of FAHW (Harvey and Hubbard 2013).

Previous research has highlighted this ‘market-failure’ for FAHW (Harvey and Hubbard 2013), and issues surrounding the free-rider incentive often associated with public goods^4^ (Uehleke and Hüttel 2019). More labelling does not seem to be the answer (Uehleke and Hüttel 2019), with it not thought to prevent true market-failure (Lusk 2011), because information on labels is not always relevant or trusted (Verbeke 2005), or there is a lack of knowledge preventing understanding of labels that are available (Norwood and Lusk 2011). However, lack of sufficient labelling has been cited as one of several barriers to purchasing HWP (Clark et al. 2016), with products from higher welfare systems not always easily distinguished in the marketplace by consumers (Buller and Morris 2004). HWP therefore do not always provide added value for producers (Buller and Morris 2004).

Finally, a lack of perceived responsibility by consumers is also important. Studies have shown that consumers allocate less responsibility for FAHW to themselves (Vanhonacker et al. 2010), and more to farmers and the government (Clark 2018). The aforementioned cited barriers to the purchase of HWP such as price and availability, along with the private and public good benefits that ensuring FAHW offers (Lusk and Norwood 2011; Bennett et al. 2012), highlight the need for regulatory and policy actions (McVittie et al., 2006). The provision of increased FAHW via taxpayer funding as a public good could be a viable solution to provide FAHW at acceptable levels, with subsidies helping to offset any increased costs incurred by producers. Given that taxpayers’ money is being proposed to fund such an approach it is thus important to gauge public perceptions of the issue. This paper therefore looks to explore the degree of public support for a public goods approach to FAHW, using England as a case study. We also explore consumer perceptions of mechanisms for presenting FAHW alternatively as a private good.

## Methods

A survey (see supplementary material) was designed to explore the UK public’s perceptions of farming, FAHW, and FAHW as a public good. The survey was built in Qualtrics survey software (Qualtrics 2021), and informed by a review of the current literature and an earlier public engagement activities including Clark and Mahon (2023), which highlighted several topics for further exploration with the public, including labelling, the role of animal welfare in food choice, and views on UK farming. These topics would also serve to add additional background information on participants in addition to their thoughts on whether FAHW should be a public good.

Given the timing of the survey (2020), and the focus on public goods, a short introduction to the UK Agriculture Bill was provided in the survey, accompanied with a simplified explanation of a public good to ensure that all participants understood what the concept meant:

*“A public good is something which is thought to benefit everyone and exclude no one. They are often unprofitable to produce privately. Examples of public goods are clear air, clean water and biodiversity”.*

Questions were then asked in relation to agricultural public goods, with participants asked to: (1) rate their agreement as to whether taxpayers’ money should be used to pay farmers for nine separate public goods as outlined in Defra (2021); (2) rank the top three public goods that they would prioritise farmers to deliver, and; (3) state their level of agreement with FAHW being specifically considered as a public good. This format allowed for the comparison of responses across questions in relation to how FAHW was prioritised as a public good in relation to other potential factors. Respondents were also asked about the responsibility of different stakeholders for ensuring FAHW in the UK given that this could give further indication as to how FAHW could be regulated, e.g., through consumers and the market or through the government and regulatory mechanisms, and that responsibility has previously been shown to vary across stakeholders (Clark 2018). Additional questions focused on factors important to participants in their choice of animal products whilst purchasing, including their awareness of use and trust in UK food labels associated with animal product welfare and quality standards e.g., Red Tractor, RSPCA Assured, Soil Association, Quality Standard and Lion Eggs^5^. The questions on labelling were included to provide insights into the current use of private mechanisms for indicating animal product quality elements, including FAHW. The labels included within this question were compiled using labels from government approved farm assurance schemes (e.g., Red Tractor), other national level farm assurance schemes (e.g., Quality Standard), with an organic label also included as organic standards are often associated with consumers for higher animal welfare standards (Clark et al. 2016). All questions were asked on 5-point Likert scales, other than for awareness of labels which was a yes/no response question, and the ranking question for public goods.

The survey was piloted with 12 members of the public to check survey length, question order and comprehensibility in June 2020. Ethical approval was obtained prior to survey distribution (Reference 3434/2020). A representative sample (in relation to age and gender) of the UK population was obtained through use of a Qualtrics panel, with sampling and distribution organised by Qualtrics. Qualtrics panellists are self-selecting and are vetted upon sign up to the panel. All survey responses using the panel have checks inbuilt to help with data quality including bot response prevention, speeding and geo-IP restriction. Survey respondents were matched for age and gender based on 2001 UK census data. Online panels are noted not to be as good as probabilistic random samples from the target population. They have been noted as having issues with data quality, including in relation to comprehension, attention, reliability, honesty, (Peer et al. 2022; Scherpenzeel 2018). However, research has also indicated that in-person and online panel responses do not differ in terms of sampling performance, although the researchers in this study did note issues with speeders (Sandorf et al. 2016). In addition to the automated checks, steps were taken by the research team to minimise potential data quality issues. These included removing responses (*n* = 101) that failed to reach a minimum time to complete based on the pilot survey, duplicate responses, open-ended question responses that did not make sense in relation to the questions, and participants who gave the same response to all questions (Hays et al. 2015), leaving a sample of 521 valid survey responses. The survey was run online between 5th October and 1st November 2020. Data were exported into Stata for analysis (StataCorp 2021). Descriptive statistics and an ordered logistic regression were then conducted to explore the effects of socio-demographic characteristics, responsibility and support for FAHW as a public good. A marginal effects analysis was then conducted to visualise the heterogeneity in the data for selected significant variables from the ordered logistic regression.

## Results

### Participant overview

Table 1 provides an overview of participant characteristics for the 521 respondents. The sample is largely representative of the population based on age and gender, although older individuals are slightly underrepresented, which is common for online panels (Scherpenzeel 2018).Around three quarters of participants (76.2%) ate omnivorous diets, with the rest describing themselves as flexitarian, vegetarian or vegan. These values are slightly higher than those reported previously (Stewart et al. 2021). This could be due to the topic of the survey and resultant selection bias, with those with an interest in the subject matter more likely to take part.

**Table 1:** Respondent characteristics

Participant Characteristics	Number of respondents (%)	2021 UK Census (%)*
**Age**		
18–24 years	61 (11.7)	6.04**
25–34 years	102 (19.6)	13.51
35–44 years	94 (18.0)	12.98
45–54 years	103 (19.8)	13.28
55–64 years	87 (16.7)	12.56
65 + years	74 (14.2)	18.56
**Gender**		
Man	251 (48.2)	48.96
Women	267 (51.2)	51.04
Genderqueer or non-binary	3 (0.6)	***
**Dietary choices**		
I eat meat and plants (omnivore)	397 (76.2)	97.7
I am a flexitarian	72 (13.8)	-
I am a vegetarian	33 (6.3)	2.1
I am a vegan	11 (2.1)	0.2
I do not wish to specify	8 (1.5)	

*Age and sex information taken from 2021 UK census data (Office for National Statistics 2022). Dietary information taken from (Stewart et al. 2021)

**Data taken from the 20–24-year-old 5-year age bracket

***information not provided

### Understandings of government policy on agriculture and public good priorities

Many participants (75.4%) did not know that a new Agriculture Bill had been proposed^6^. 72.0% of respondents agreed (strongly agree/agree) that FAHW should be considered as a public good when questioned directly (Table 2). Participants were also asked whether they would support the use of taxpayers’ money to pay farmers to deliver public goods (Table 3). A slightly higher percentage disagreed that they would be WTP for FAHW as a public good (7.1% Table 3) compared to disagreeing that it should be considered a public good (6.3% Table 2), with a higher percentage also responding, ‘neither disagree nor agree’ (20.9% vs. 15.2%) to the WTP question.

**Table 2:** The extent to which respondents (*n* = 521) agreed or disagreed that farm animal health and welfare should be considered as public goods. Reported as number (%)

	Strongly disagree	Disagree	Neither disagree nor agree	Agree	Strongly agree
Farm animal health and welfare should be considered as public goods	11 (2.1)	26 (5.0)	109 (20.9)	217 (41.7)	158 (30.3)

When presented with a list of possible public goods from agriculture, support for all was strong (Table 3) with all having a majority agreement (agree/strongly agree), and less than 15% of participants disagreeing (disagree/strongly disagree). ‘Farm animal health and welfare’ (78.5% agree/strongly agree), ‘Clean and plentiful water’ (75.9% agree/strongly agree) and ‘Soils managed sustainably’ (75.4% agree/strongly agree) were the three highest scoring. For all goods listed, between 15 and 30% of participants responded, ‘neither disagree nor agree’.

**Table 3:** The extent to which respondents (*n* = 521) agreed or disagreed with the use of taxpayers’ money to pay farmers to deliver each public good. Reported as number (%)

Public Good*	Strongly disagree	Disagree	Neither disagree nor agree	Agree	Strongly agree
Farm animal health and welfare	13 (2.5)	20 (3.8)	79 (15.2)	189 (36.3)	220 (42.2)
Clean and plentiful water	18 (3.5)	15 (2.9)	93 (17.9)	228 (43.8)	167 (32.1)
Thriving plants and wildlife	17 (3.3)	16 (3.1)	103 (19.8)	228 (43.8)	157 (30.1)
Tackle climate change	25 (4.8)	23 (4.4)	120 (23.0)	217 (41.7)	136 (26.1)
Soils managed sustainably	18 (3.5)	19 (3.6)	91 (17.5)	261 (50.1)	132 (25.3)
Reduced risks from hazards e.g., flooding	17 (3.3.)	18 (3.5)	114 (21.9)	237 (45.5)	135 (25.9)
Enhancing beauty and heritage	14 (2.7)	40 (7.7)	148 (28.4)	213 (40.9)	106 (20.3)
Clean air	25 (4.8)	40 (7.7)	122 (23.4)	234 (44.9)	100 (19.2)
Engagement with the public	18 (3.5)	47 (9.0)	136 (26.1)	228 (43.8)	92 (17.7)

*Public goods taken from Defra (2021)

### Responsibility for farm animal health and welfare

Participants were asked who they thought should be responsible for FAHW (Table 4). The highest agreement (agree/strongly agree) was for farmers (83.3%), with half (49.9%) strongly agreeing that it was farmers’ responsibility. This was followed by the government (76.4%), and vets (75.0%). ‘You as a consumer’ and ‘the general public’ were the groups associated with the least responsibility (65.3% and 59.4%, respectively, agreed or strongly agreed), although more than half still agreed that these groups have some responsibility. It should be noted that there was very little disagreement for any of the stakeholders listed (less than 10% disagree/strongly disagree).

**Table 4:** The extent to which respondents (*n* = 521) agreed or disagreed that different stakeholders should be responsible for ensuring the health and welfare of farm animals in the UK. Reported as number (%)

Stakeholder	Strongly disagree	Disagree	Neither agree nor disagree	Agree	Strongly agree
Farmers	2 (0.4)	13 (2.5)	72 (13.8)	174 (33.4)	260 (49.9)
The government	5 (1.0)	23 (4.4)	95 (18.2)	231 (44.3)	167 (32.1)
Quality assurance systems	4 (0.8)	13 (2.5)	134 (25.7)	223 (42.8)	147 (28.2)
Animal welfare organisations	9 (1.7)	24 (4.6)	100 (19.2)	243 (46.6)	145 (27.8)
Food manufacturers	3 (0.6)	27 (5.2)	111 (21.3)	237 (45.5)	143 (27.4)
Vets	2 (0.4)	26 (5.0)	102 (19.6)	256 (49.1)	135 (25.9)
Abattoirs	14 (2.7)	26 (5.0)	175 (33.6)	180 (34.5)	126 (24.2)
Food retailers	3 (0.6)	34 (6.5)	129 (24.8)	229 (44.0)	126 (24.2)
You as a consumer	8 (1.5)	32 (6.1)	141 (27.1)	235 (45.1)	105 (20.2)
The general public	8 (1.5)	40 (7.7)	164 (31.5)	217 (41.7)	92 (17.7)

### Animal welfare and food purchasing

Respondents who consumed animal products were asked to rate the importance of several factors in their purchasing decisions (Table 5). Taste (86.6% very important/important), food safety (81.8%) and price (77.9%) were the three most important factors, with animal welfare ranking fourth (72.6%). All factors, except whether the food was organic, were very important/important for more than half of respondents.

**Table 5:** The importance of different factors in respondents’ choice of animal products. Reported as number (%)*

	Very unimportant	Unimportant	Neither unimportant nor important	Important	Very Important	Total responses
Animal welfare	24 (4.6)	25 (4.8)	74 (14.2)	215 (41.3)	163 (31.3)	501(96.2)
Country of origin	25 (4.8)	41 (7.9)	123 (23.6)	226 (43.4)	91 (17.5)	506 (97.1)
Environmental impact	23 (4.4.)	34 (6.5)	128 (24.6)	217 (41.7)	106 (20.3)	508 (97.5)
Food safety	16 (3.1)	13 (2.5)	47 (9.0)	178 (34.2)	248 (47.6)	502 (96.4)
Health	12 (2.3)	19 (3.6)	109 (20.9)	229 (44.0)	132 (25.3)	501 (96.2)
Locally sourced	23 (4.4)	40 (7.7)	154 (29.6)	215 (41.3)	76 (14.6)	508 (97.5)
Organic	56 (10.7)	90 (17.3)	185 (35.5)	129 (24.8)	47 (9.0)	507 (97.3)
Price	7 (1.3)	10 (1.9)	8 (15.5)	249 (47.8)	157 (30.1)	504 (96.7)
Taste	9 (1.7)	6 (1.2)	37 (7.1)	188 (36.1)	263 (50.5)	503 (96.5)

*This question was not answered by respondents who identified as vegan. In addition, 10 other respondents did not provide answers for all factors listed

Respondents were also asked to indicate their awareness of several food labels associated with animal products in the UK. Table 6 highlights that Lion Eggs (80.8% Yes) and Red Tractor (73.9% Yes) are those most recognised with Soil Association the least (56.4% No).

**Table 6:** Responses to whether participants (*n* = 521) had heard of UK food labels. Reported as number (%)

	Yes	No
Red Tractor	385 (73.9)	136 (26.1)
RSPCA Assured	277 (53.2)	244 (46.8)
Soil Association	227 (43.6)	294 (56.4)
Lion Eggs	421 (80.8)	100 (19.2)
Quality Standard	313 (60.1)	208 (39.9)

When asked to rate their trust in the same labels, Lion Eggs was the most trusted (Table 7). Nearly all of those listed were trusted by more than half of participants (some/a lot of trust in them), other than the Soil Association (48.4% some/a lot of trust in them).

**Table 7:** Participants (*n* = 521) trust in food labels. Responses reported as number (%)

	I have no trust in them	I have a little trust in them	I neither distrust nor trust them	I have some trust in them	I have a lot of trust in them
Red Tractor	10 (1.9)	64 (12.3)	171 (32.8)	168 (32.3)	108 (20.7)
RSPCA Assured	16 (3.1)	50 (9.6)	168 (32.2)	172 (33.0)	115 (22.1)
Soil Association	11 (2.1)	42 (8.1)	216 (41.5)	176 (33.8)	76 (14.6)
Lion Eggs	9 (1.7)	46 (8.8)	132 (25.3)	176 (33.8)	158 (30.3)
Quality Standard	17 (3.3)	44 (8.4)	155 (29.8)	179 (34.4)	126 (24.2)

### Variation in support for taxpayer funding of FAHW

An ordered logistic model was estimated to examine the association between a respondent’s support for the use of taxpayer money to fund payments to farmers to deliver higher FAHW standards and a range of potential explanatory factors. This type of model allows for the analysis of the relationship between different independent variables and a categorical dependent variable. Several factors shown to influence perceptions of FAHW were included in the model: the sociodemographic characteristics age, gender, income, education; dietary preference, place of education (Clark et al. 2016, 2017), perceptions about the responsibilities of key stakeholders (Clark 2018), the importance of FAHW in food choice (Janssen et al. 2016), and perceptions about the public good attributes of FAHW.

From Table 8, respondents who agreed that animal welfare organizations, farmers, the government, quality assurance systems, and consumers, are responsible for FAHW in the UK are more likely to support taxpayer funding of FAHW. Support is also higher amongst respondents who consider FAHW to be a public good. As compared to the oldest segment of respondents (65 + years), younger respondents (ages 18–24 and 35–44) are less likely to support the use of taxpayer money to support FAHW in the UK.

**Table 8:** Estimates of multivariate ordered logit regression

Variables	Coefficient estimate	z
**Institutions responsible for FAHW in the UK**		
Animal welfare organizations e.g., RSPCA	0.26**	2.00
The government	0.52***	3.99
Veterinarians	0.01	0.10
Farmers	0.57***	3.66
Abattoirs	-0.01	-0.15
Quality assurance systems	0.26*	1.80
Food manufacturers	-0.05	-0.31
Food retailers	-0.03	-0.23
The general public	-0.19	-1.21
You as a consumer	0.49***	3.17
***Geographical distribution***		
England	-0.19	-0.44
Northern Ireland	0.10	0.14
Scotland	-0.56	-1.04
***Dietary choice***		
Non-omnivores	-<0.01	-0.01
***Socio-demographic factors***		
Female	0.30	1.48
Rural	-0.26	-1.16
University degree	-0.06	-0.27
Age (18–24 years)	-0.92**	-2.29
Age (25–34 years)	-0.35	-0.96
Age (35–44)	-1.02***	-2.94
Age (45–54)	-0.79**	-2.30
Age (55–64)	-0.63*	-1.78
*Income*		
<£10,000	0.13	0.27
£10,001 - £20,000	-0.01	-0.03
£20,001 - £30,000	-0.07	-0.15
£30,001 - £40,000	-0.13	-0.28
£40,001 - £50,000	0.35	0.71
£50,001 - £60,000	0.78	1.45
>£60,000	0.21	0.42
***Other factors***		
Importance of animal welfare in food choice	0.16	1.73
Agree that FAHW is public good	0.43***	3.43
N	501	
LR chi2 (31)	240.68	
Prob > Chi2	0.00	
Log Likelihood	-491.00	
Pseudo R2	0.20	

*Notes* ***1%; ** 5%; and, * 10% significance level. Dependent variable is the support for the use of taxpayers’ money to pay farmers to deliver FAHW. Baseline year = 65 + years; income for respondents who chose not to report income omitted; dummy variable for Wales omitted due to collinearity

**Fig. 1:**
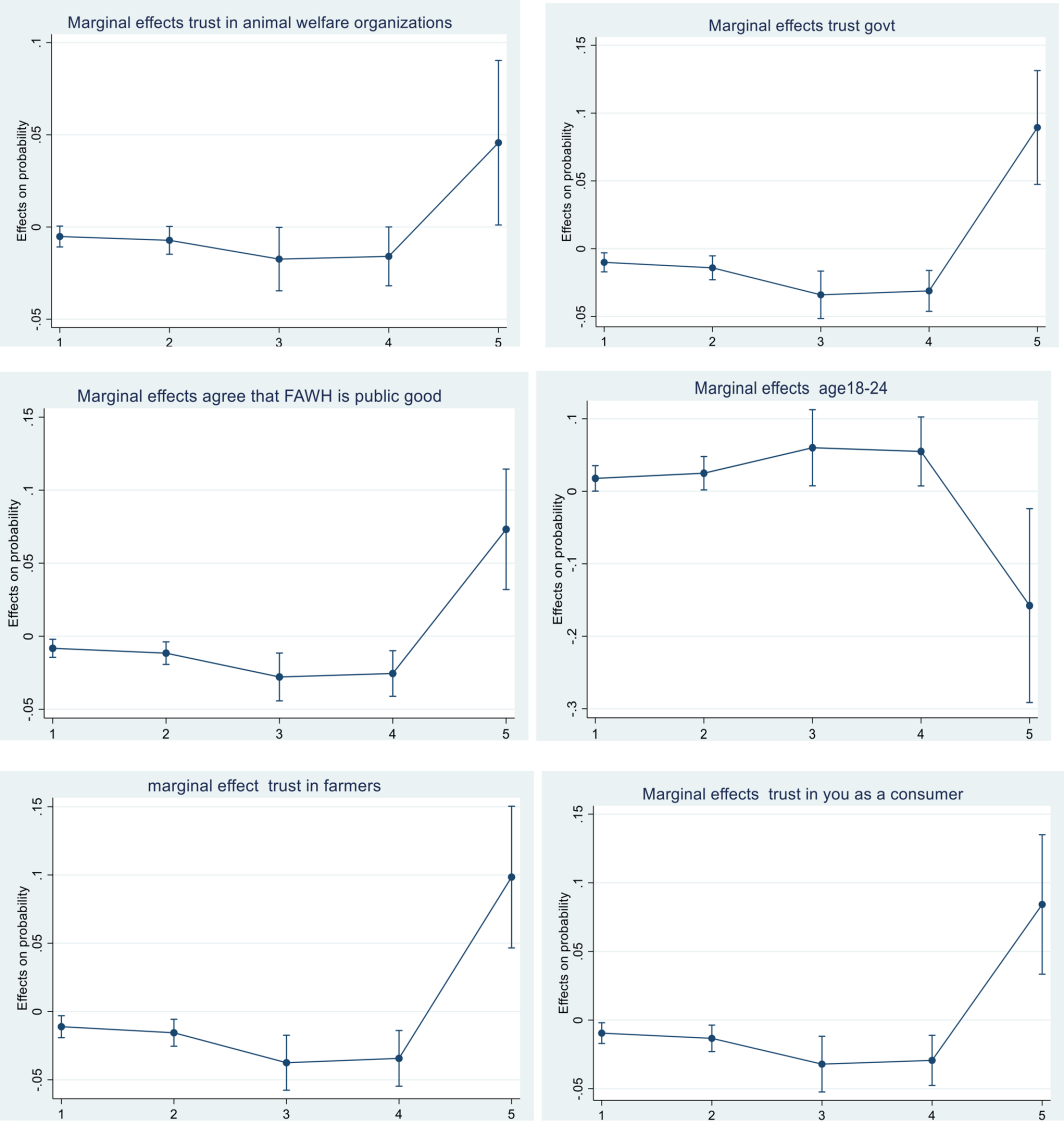
This Figure shows the trends in marginal effects for selected perceptions and demographic variables. Horizontal axis is the is the support for the use of taxpayers’ money to pay farmers to deliver farm animal health and welfare (FAHW) (1 = strongly disagree;5 = strongly agree). Error bars represent 95% confidence interval

The marginal effects of several parameters (variables) included within the ordered logistic model were also explored to look for potential heterogeneity in the data (Fig. 1). Marginal effects look to explore the partial derivative of a variable. They do this by looking to see the effect of a change in one variable when all other variables are held constant. It therefore allows you to see how much one variable will impact the outcome, in this case the outcome being support for farmers to deliver FAHW as a public good, by trying to isolate its effects from the other variables in the model.

The variables chosen for exploration with marginal effects were those that were shown to be statistically significant in the ordered logistic model (Table 8). This analysis shows evidence of heterogeneity in the predictive probabilities of the different variables analysed (see Fig. 1), meaning that the different parameters have variable effects on support for farmers to deliver FAHW as a public good. Figure 1 shows the effects on probability for each variable across each of the 5 Likert scale responses. The higher the probability, the more likely respondents are to give that particular response on the Likert scale. In the context of our analysis, these predicted probabilities are derived from the marginal effects of the independent variables included in our model. Marginal effects help us understand how changes in these variables impact the likelihood of respondents providing specific responses on the Likert scale. For example, an increase in the marginal effect of a certain variable leads to a higher predicted probability of respondents agreeing or strongly agreeing with a statement on the Likert scale, while a decrease results in a lower probability of agreement. In Fig. 1, respondents who agree that FAHW is a public good are 7.3% more likely to strongly agree with the use of taxpayers’ money to pay farmers to deliver FAHW compared to 4.5% for those who trust in animal welfare organisations. In contrast, respondents in the 18–24 age category were 15.7% less likely to strongly support the use of taxpayers’ money to pay farmers to deliver FAHW.

## Discussion

These results shed new light on public attitudes towards incorporating FAHW within the ‘public money for public goods’-based approach informing agricultural policy. These novel insights on public support for FAHW as a public good, perceived responsibility and the use of labelling and implications for market-based solutions are discussed in the following sections.

### Support for FAHW as a public good

Although there was an overall lack of awareness of the new Agriculture Bill (now Act), participants had strong views on what should be considered as public goods and the use of taxpayers’ money to fund them. Findings show support for the inclusion of a range of outputs as public goods, aligning with previous research that demonstrated public support for farmers to be compensated for the provision of public goods (e.g., environmentally friendly farming) should their provision cost more than current farming practice (Howley et al. 2014).

Of all the public goods listed FAHW received the highest level of agreement and support for public funds to enable it. There was less support for FAHW as a public good amongst younger participants. Whilst previous research has highlighted that younger individuals typically have higher concern over FAHW, there is some evidence indicating that younger people tend to be less supportive of food policies in several countries including the UK (Kwon et al. 2019) and are less trusting of government intervention (Diepeveen et al. 2013). The findings presented here may be indicative of the fact that younger respondents prefer other mechanisms such as advocacy as opposed to taxation, for example. More research is needed understand the trade-offs between concern for FAHW and preferences for specific regulatory mechanisms.

Overall, FAHW would therefore appear to be a priority for members of the public, although all the potential public goods listed were supported by more than 60% of the sample. Whilst the prioritisation of FAHW could be related to the topic of the survey with sheep and cattle farming clearly included with the survey pre-information for all participants, previous European research (Uehleke and Hüttel 2019) and consultations by Defra (2018) also found support for the provision of high levels of FAHW as a public good from a range of stakeholders, although the responses of the general public were not presented as a separate group within the Defra research. Our findings provide evidence of public support for taxpayers’ money being used in this way to support farmers in England to deliver FAHW through the AHWP.

### Responsibility and market solutions

Whilst all stakeholders were perceived as responsible – to some degree – for ensuring FAHW, as per previous research (Thorslund et al. 2017), farmers were regarded as having the greatest responsibility, followed by the government. Consumers and members of the wider public were seen to have the least overall responsibility as identified previously (Clark 2018). Results of the regression analysis also identified that those who believed farmers, government and consumers had responsibility were more likely to support taxpayer funding. Therefore, if consumers do not view themselves as responsible, consumer beliefs or actions are unlikely to drive change – either through their support for public goods or through altering their purchasing habits towards HWP.

Market-failure will continue to occur given the inability of the market to fully recoup the costs generated through improved FAHW delivery, and issues with the free-rider problem (see, Lusk (2011) for a discussion). Previous research has highlighted a growing concern over FAHW and subsequently strong preferences and WTP by the public for higher FAHW (Clark et al. 2017; Lagerkvist and Hess 2011) and higher animal health products (as distinct and separable from HWP) (Rodrigues and Hanley 2021). Yet there is a noted disconnect between *stated* purchase intentions and *actual* purchasing behaviour (Clark et al. 2016; Malley and Southam 2018). Although we did not measure WTP or present participants with a trade-off, our findings would appear to reflect this with animal welfare being considered an important attribute whilst purchasing for most participants, yet recognition of higher welfare labels being considerably lower, suggesting that consumers may not be using labels to inform their purchasing. This information asymmetry therefore means consumers cannot make informed decisions, so that farmers are unable to recoup all cost increases from delivering higher welfare-products from the market. Whilst criticism of animal welfare labelling exists (Uehleke and Hüttel 2019), it would appear that more effective means of communication, either on packaging, or more widely at the point-of-purchase and increasingly beyond, is needed to increase the information available to consumers, especially with FAHW not always salient to consumers at the point-of-purchase (Malley and Southam 2018). The findings support a need to better engage with consumers on FAHW issues including more research to identify their specific information needs and requirements (Verbeke 2005).(RSPCA et al., 2023, Connors et al. 2022; YouGov 2022).

### Delivering a public goods approach to FAHW

Previous research (Uehleke and Hüttel 2019) has shown that whilst the market can be effective to some extent (Thorslund et al. 2017) it is not sufficient by itself to deliver the improvements to FAHW that proponents of market-based solutions hope for (see Esbjerg (2020) for a more in-depth discussion), and is not successful in delivering a sufficient quantity of public goods (Renard 2005; Busch 2014) such as FAHW (Hubbard et al. 2020; Harvey and Hubbard 2013). The democratic legitimacy of market-based approaches has also been questioned in relation to accountability, transparency and participation (Fuchs et al. 2011), although initiatives such as the Business Benchmark for Farm Animal Welfare^7^ aim to change this in relation to some of the aforementioned criticisms, in particular around transparency (McLaren and Appleyard 2019). The findings here identified public support for FAHW measures, implying that a public money for public goods approach within the new Agriculture Bill may well be an appropriate as well as publicly-supported, intervention, particularly if suitable *de minimis* standards are in place.

Overall, our findings support a need for greater food democracy, in particular in relation to several of the characteristics highlighted by Petetin (2016), including the need for more accurate information and choice being offered to consumers, and a bottom-up approach to the decision-making process. The latter point is particularly pertinent given topics where values are considered, such as public goods like FAHW, and the role of consumer responsibility in supporting funds being spent on FAHW. There needs to be collective debate involving all stakeholders, including the public, over what standards of FAHW are acceptable, and consideration of how public views of acceptable FAHW may evolve (Hassanein 2003). Part of this includes having broader conversations surrounding food and farming as a means of meeting consumers’ information needs, reducing the growing disconnect between food production and consumption, with previous engagement work by the authors highlighting an interest in this (Clark and Mahon 2023). This may also give rise to other means of individuals expressing their wishes, such as advocacy, ballots, or not-for-profit campaigning that have been shown to be effective in providing support for public goods (Grant and Langpap 2019).

Given high levels of concern for FAHW within the UK and further afield (Clark 2018; European Commission 2016; Lusk et al. 2007), and support for FAHW inclusion as a public good in this survey, supporting the delivery of high FAHW standards through the use of taxpayers’ money could be an important step in ensuring that the market meets the demands and needs of consumers and the wider public, whilst maintaining affordable pricing (Ingenbleek et al. 2012; McInerney 2004). This is particularly pertinent given the importance of price to the majority of respondents in their purchasing decisions, and those in previous research (Malley and Southam 2018; Eurobarometer 2019), with cost often cited as a barrier to purchasing HWP (Clark et al. 2016). Amidst an ongoing cost of living crisis in the UK food prices are a growing concern (Connors et al. 2022). Whilst those on higher incomes maintain more agency over purchasing including purchasing food that aligns with their values (Connors et al. 2022)other consumers are trading down to lower quality items with perceived lower standards of animal welfare (YouGov 2022). There is also a shift away from consuming animal products (RSPCA et al., 2023, YouGov 2022). Yet animal welfare remains a priority with strong public support (RSPCA et al., 2023). This reinforces the need for strong welfare standards that all those who can afford to.

The use of taxpayers’ money would also ensure that farmers are financially supported in providing socially-acceptable levels of FAHW, and this is especially important given that labelling/assurance programmes do not often guarantee farmers’ financial compensation for the costs of measures needed to improve FAHW over a time span sufficient for recouping of the costs (Sørensen and Schrader 2019). This is particularly relevant given the rising production costs within the sector (Riley 2022) and power imbalances and complexity within livestock supply chains due to increased expansion and consolidation of food retailing and accompanying backwards vertical integration of supply chains (Richards et al. 2013; Fearne 1998). Moreover, research with farmers supports this approach, with farmers favouring the provision of public goods, providing that they are financially remunerated (Cusworth and Dodsworth 2021).

## Conclusion

This paper has considered the shift in policy support for FAHW as part of a wider public goods approach to agricultural support in England and offered novel insights on the public’s response to this. As food democracy rests on the principle that every citizen has a contribution to make in negotiating how food is provided within a society (Hassanein 2003), there is a need for improved engagement with the public during policymaking, including more creative and inclusive mechanisms (Clark and Mahon 2023), to maximise opportunities for the public to be informed about and input into changing legislation, such as discussions of what should count as a public good (Hejnowicz and Hartley 2018). This may also help individuals to see the influence they can exert on the food system, including through their food choices (Booth and Coveney 2015), contributing to political decision making or through advocacy. These results make an important contribution to these debates and offer new insights for regions considering similar approaches to FAHW management.

The high percentage of ‘neither disagree nor agree’ responses to questions, and low recognition of and trust in existing food labels would suggest, however, that there is a lot that the public do not know or understand about farming and government support for farming, and/or there are some members of the public who do not feel as strongly about these issues. It also indicates a need for more engagement and transparency surrounding both the public and private provision of FAHW. Of note in this research was that participants’ willingness to support FAHW provision using taxpayers’ money was slightly lower than their agreement for whether FAHW should be considered as a public good. Future research should look to explore this disconnect further, and possible reasons behind this.

Indeed, the need for greater transparency in food production is emphasised in the Agriculture Transition Plan (Defra 2020a), for English agricultural policy. Greater transparency is therefore important in showing how production standards between products are different, e.g., certification/labelling on housing systems, and how decisions over what concerns have been incorporated into legislative *de minimis* standards have been made, and what exactly they involve, so as to enable informed consumer choice and provide an effective public policy approach (Vanhonacker and Verbeke 2014; Lagerkvist and Hess 2011). This also aligns with wider strategies to ensure safe and trusted food systems (Food 2022), and creating a more transparent food system to help consumers better understand where the food they consume comes from (Defra 2022b). A more informed public may also help reduce the information asymmetries that contribute towards market-failure.

Whilst this research presents several novel findings there are limitations of the approach that should be taken into consideration. Firstly, the timing of the survey during the Covid-19 outbreak may have affected individuals’ food purchasing behaviours and prioritisation of different animal product attributes (Food 2021). The growing discussions around human health (and the transmission of disease from animals to humans) may also have influenced individuals’ views. Secondly, whilst a definition of public goods was provided in the survey to aid participants in completing the survey, qualitative questions to explicitly explore how they understood or interpreted the term were not asked. Future research could explore qualitatively what the public understands by the term public good, how this influences their views of what should be provided through both market-based and policy mechanisms, including what this might mean in practice for different animals in different farming systems.

## Data Availability

The data that support the findings of this study are available from the corresponding author upon reasonable request.

## Footnotes

1 Although we recognise that some may consider health and welfare as two separate concepts, and others as a single concept, this paper treats the term as a single one for ease of participant understanding in the survey.

2 See Uehleke and Hüttel (2019) for an overview.

3 Sandøe and Christensen (2018 highlight several possible reasons for this including: accession of several Eastern European countries to the EU with fewer concerns about animal welfare, a focus on dealing with economic crises in several countries, growing scepticism about the EU from member countries, and increasing competition from producers outside the EU.

4 The free-rider issue is a potential problem when markets rather than government provide public goods. Free-riding exists when individual actions (i.e., purchasing) generate too little of the public good compared to the optimal amount that could be delivered (i.e., through co-ordinated/collective efforts such as taxation).

5 An overview of these different labels can be found here: https://www.bva.co.uk/media/3767/bva-uk-farm-assurance-schemes-choose-assured-infographic-october-2020.pdf.

6 For context, the survey was run between 5th October − 1st November 2020 with the Agriculture Bill raised on 16th January 2020 and passing into law on the 11th November 2020.

7 The Business Benchmark for Farm Animal Welfare analyses and ranks the world’s leading food companies on their farm animal welfare policies and commitment, based on publicly available information.
